# Elephant barrier behaviors in response to conflict mitigation fences

**DOI:** 10.1111/cobi.70258

**Published:** 2026-03-19

**Authors:** Dominique Gonçalves, Robert J. Smith, Helen M. K. O'Neill

**Affiliations:** ^1^ Durrell Institute of Conservation and Ecology (DICE) University of Kent Canterbury UK; ^2^ Gorongosa National Park Scientific Department Sofala Mozambique; ^3^ Department of Ecology and Evolutionary Biology Princeton University Princeton New Jersey USA; ^4^ Institute of Zoology Zoological Society of London London UK

**Keywords:** animal movement, barrier behavior analysis, beehive fences, elephants, human–wildlife conflict, landscape conservation, mitigation, análisis de comportamiento ante barreras, cercas de colmena, conflicto humano‐fauna, conservación del paisaje, elefantes, mitigación, movimiento animal

## Abstract

Human–wildlife conflict is a major conservation issue, particularly in lower income countries, where it affects marginalized people and leads to the extirpation of threatened species. Managers increasingly use fences to reduce this conflict but lack evidence on the effectiveness of these barriers, especially on whether this reduces the number of incidents or only shifts the problem elsewhere. We adapted an approach designed to measure how individual animals respond to barriers (barrier behavior analysis) to evaluate a human–wildlife conflict intervention. We used movement data from 20 GPS‐collared elephants to assess the extent to which their behavior was influenced by community‐managed beehive fences around Gorongosa National Park in Mozambique. We measured the number of times elephants were stopped by the fences and compared this with the number of times elephants were stopped by a natural barrier formed by a major river. Beehive fences blocked elephant movement in 69.3% of encounters, whereas the river barrier blocked 35.9%. Human–elephant conflict levels were lower after construction of the fence, dropping from a mean of 566 crop and infrastructure damage incidents per year in 2018 and 2019 to a mean of 117.5 incidents per year in 2020 and 2021. The mean distance of crop and infrastructure damage incidents from the park boundary increased from 0.98 to 1.97 km, and the number of human injuries and deaths increased from 1 to 8. Our results showed that community‐run beehive fences can be effective barriers and reduce overall levels of human–elephant conflict in agricultural landscapes. They also showed how fencing can change the spatial pattern of conflict. This highlights the benefits of understanding how conflict mitigation methods change individual animal behavior and of measuring intervention effectiveness at a landscape scale.

## Abstract (es)

Comportamiento de elefantes ante barreras como respuesta a los cercos de mitigación de conflictos

**Resumen:**

El conflicto humano‐fauna es un problema importante para la conservación, especialmente en los países de bajos ingresos donde afecta a las personas marginadas y provoca la extinción de especies amenazadas. Los gestores utilizan cada vez más vallas para reducir este conflicto, pero carecen de pruebas sobre la eficacia de estas barreras, especialmente sobre si reducen el número de incidentes o solo desplazan el problema a otros lugares. Adaptamos un enfoque diseñado para medir cómo responden los animales individuales a las barreras (análisis del comportamiento ante las barreras) para evaluar una intervención en el conflicto entre los seres humanos y la fauna silvestre. Utilizamos datos de movimiento de 20 elefantes con collares GPS para evaluar en qué medida su comportamiento se veía influido por los cercos de colmenas gestionadas por la comunidad alrededor del Parque Nacional de Gorongosa, en Mozambique. Medimos el número de veces que los elefantes se detuvieron ante los cercos y lo comparamos con el número de veces que se detuvieron ante una barrera natural formada por un río importante. Los cercos de colmenas bloquearon el movimiento de los elefantes en el 69.3% de los encuentros, mientras que la barrera fluvial lo bloqueó en el 35.9%. Los niveles de conflicto entre humanos y elefantes disminuyeron tras la construcción de la valla, pasando de una media de 566 incidentes de daños a cultivos e infraestructuras al año en 2018 y 2019 a una media de 117.5 incidentes al año en 2020 y 2021. La distancia media de los incidentes de daños a cultivos e infraestructuras desde el límite del parque aumentó de 0.98 km a 1.97 km, y el número de humanos heridos y muertos aumentó de 1 a 8. Nuestros resultados demostraron que los cercos de colmenas gestionadas por la comunidad pueden ser barreras eficaces y reducir los niveles generales de conflicto entre humanos y elefantes en los paisajes agrícolas. También demostraron cómo las vallas pueden cambiar el patrón espacial del conflicto. Esto pone de relieve las ventajas de comprender cómo los métodos de mitigación de conflictos cambian el comportamiento individual de los animales y de medir la eficacia de las intervenciones a escala paisajística.

## INTRODUCTION

Global biodiversity is under threat from a range of anthropogenic pressures, including land‐use change and direct persecution of species (Ripple et al., 2016). Large mammals are particularly vulnerable because they require access to extensive areas to meet their resource requirements (Ceballos et al., 2015) and often have slow population growth rates (Harris et al., 2008). Protected areas (PAs) are important for the conservation of these species because they can act as refuges from anthropogenic pressures (Joppa & Pfaff, 2011), but many PAs are not large enough to maintain viable populations. Thus, wide‐ranging species often rely on accessing habitats outside PAs to fulfil their resource requirements (Evans et al., 2019). For example, only 23% of the current distribution of cheetahs (*Acinonyx jubatus*) is in PAs (Durant et al., 2017). For Asian elephants (*Elephas maximus*), PAs often comprise less than half of individuals’ home ranges (De La Torre et al., 2022). This is also the case for both species of African elephants (*Loxodonta africana* and *Loxodonta cyclotis*), which rely on accessing parts of the landscape outside PAs to obtain the resources they need, particularly in seasonal systems (Buchholtz et al., 2020; Douglas‐Hamilton et al., 2005; Hoare, 2000, 2015).

Moving outside PAs brings large mammal species into closer proximity with humans, which can lead to a range of detrimental outcomes (Woodroffe et al., 2009). For people, this can take the form of destruction of livelihoods, risks to personal safety, fear, and trauma (Barua et al., 2013). For wildlife, this can lead to retaliatory killing and increased intolerance for wildlife and conservation efforts (Mutinda et al., 2014). Because many large mammals are keystone species, the impact of conflict goes beyond survival of wildlife populations and can affect entire ecosystems (Ripple et al., 2019).

This is the case for the African savanna elephants (*Loxodonta africana*), an endangered, keystone species. When roaming outside PAs, they are often linked to severe human–elephant conflict (HEC) (Hoare, 2000; Pringle, 2008). This HEC can negatively affect people's economic security, through crop damage and the destruction of infrastructure, and people's physical security, through dangerous encounters that can result in injury and death (Hoare, 2000; Woodroffe et al., 2009). This HEC can lead to increased hostility toward elephants, sometimes leading to retributive killing, and animosity toward conservation more generally (Barua et al., 2013).

Physical barriers are widely used by conservation practitioners as an HEC mitigation tool (Cook et al., 2018; Hoare, 2000; Osipova et al., 2018). At the most basic level, managers adopt natural barriers to define PA boundaries, for example, rivers, cliffs, and mountains, to reduce the likelihood of wild species moving outside (Cozzi et al., 2013). Fences are also widely used to prevent conflict (Hoare, 2000; Jakes et al., 2018), and electric fences reduce crop damage and other forms of HEC (Kioko et al., 2008; O'Connell‐Rodwell et al., 2000; Osipova et al., 2018; Slotow, 2012). However, electric fences are expensive and difficult to maintain (Hayward & Kerley, 2009), so conservationists are turning to alternatives. One increasingly popular approach is beehive fences, which include a beehive at regular points along the fence. When elephants try to move through the fence, they disturb the hives and are scared away by the defensive behavior of the bees (King et al., 2011). This mitigation method has the additional benefit of producing honey, which can be an important source of income and provide a tangible incentive for people to maintain their fences (Hedges & Gunaryadi, 2010; King et al., 2017; La Grange et al., 2022; Parker & Osborn, 2006).

Because HEC is a key threat to many elephant populations, numerous studies have evaluated whether fences and other mitigation barriers stop elephant movement and reduce crop loss (Buchholtz et al., 2020; Chelliah et al., 2010; La Grange et al., 2022; Naidoo et al., 2022; Parker & Osborn, 2006; Thouless & Sakwa, 1995). These studies show that crop damage associated with elephants is often seasonal and the efficiency of such barriers depends on maintenance levels, location, and spatial extent (Buchholtz et al., 2020; King et al., 2011, 2017; Osipova et al., 2018; Sitati & Walpole, 2006). However, understanding of the wider impacts of barriers is still limited. A key knowledge gap is how individual animals respond when they encounter a barrier because behaviors associated with linear barriers often vary between individuals and species (Xu et al., 2021). More research is also needed on the impacts of the displacement of individual movement behavior at a landscape scale because there is a risk of interventions being identified as successfully reducing HEC when it has only moved the conflict elsewhere (Osipova et al., 2018).

We addressed this knowledge gap through a study of Gorongosa National Park's (GNP) elephant population, which has increased substantially to over 1000 individuals (Campbell‐Staton et al., 2021) in recent years after experiencing a steep decline due to illegal poaching. In the last 5 years, HEC has been a persistent problem along the southern border of the park (Branco et al., 2020). In an attempt to reduce crop damage, park staff have worked with affected communities to implement mitigation strategies, including installing beehive fences (Branco et al., 2020). Understanding elephant behavioral responses to these fences and the associated impact on crop damage is important to help park managers adapt and prioritize coexistence techniques in the buffer zone of GNP. We investigated the movement behaviors elephants exhibit when encountering a natural barrier (the river along the southern border of GNP) and a conflict mitigation barrier (beehive fences), the factors that explain the different types of behaviors recorded, and whether HEC patterns have changed since the installation of the beehive fences.

## METHODS

### Study system

The GNP in Mozambique is a 3674‐km^2^ PA containing a range of habitat types that include miombo woodland, wooded savannas, riverine forest, and open floodplains (Stalmans & Beilfuss, 2008). The climate consists of a wet season from November to April and a dry season from May to October (Potter et al., 2022; Stalmans & Beilfuss, 2008). The park is surrounded by a 5333‐km^2^ buffer zone, which is home to 200,000 people who chiefly depend on subsistence agriculture (Branco et al., 2019).

**FIGURE 1:**
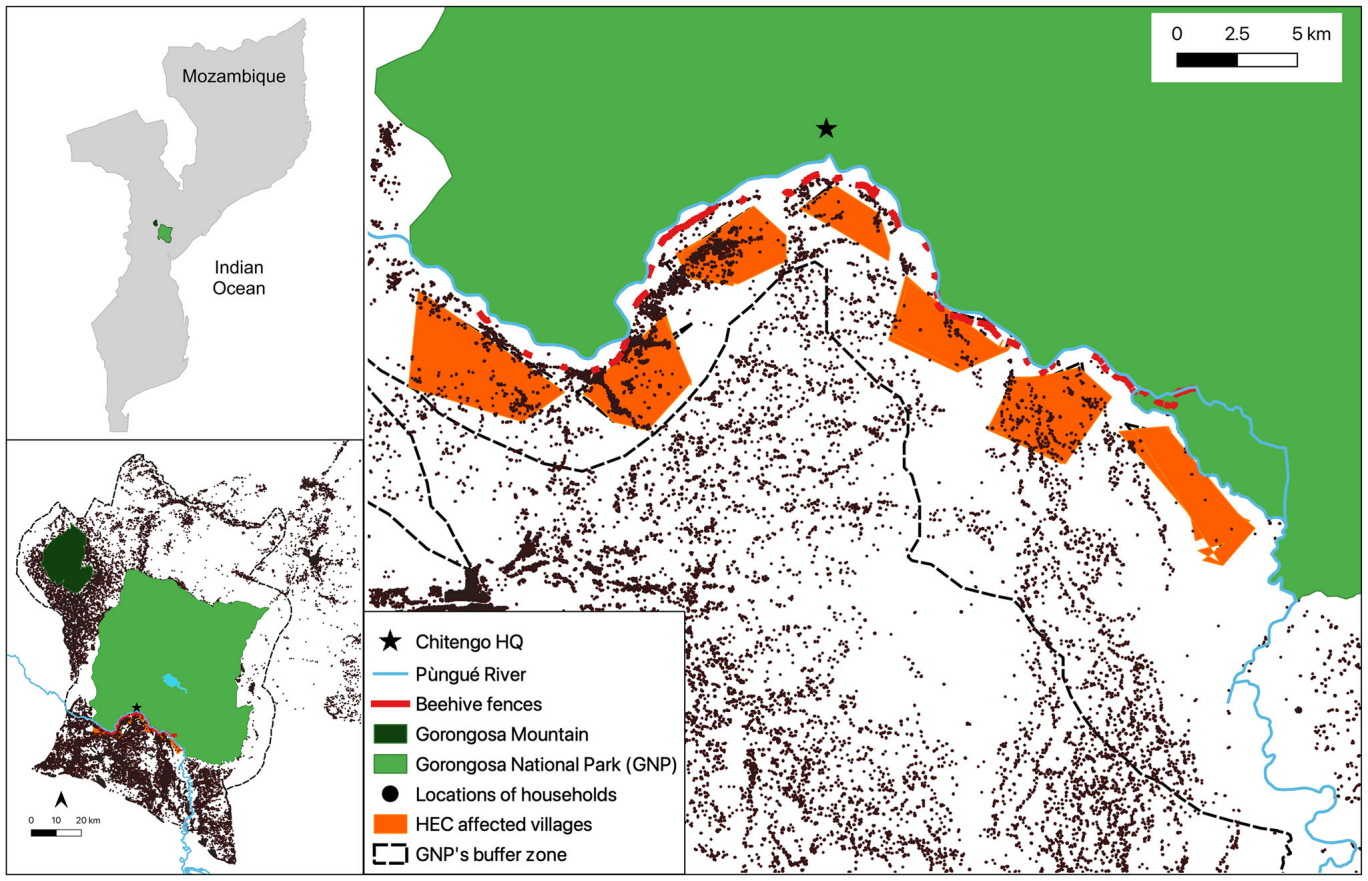
(a) Location of Gorongosa National Park (GNP) in Mozambique, (b) buffer zone in the GNP (the Pùngué River runs along the southern boundary of GNP through the buffer zone), and (c) locations of communities affected by human–wildlife conflict, beehive fences, and GNP headquarters at Chitengo.

The southern border of GNP is demarcated by the Pùngué River, and elephants frequently cross this to eat crops in the buffer zone (Branco et al, 2020). Some sections of the river are more difficult for elephants to forge because of the topology, and this is exacerbated during the wet season when the width and depth of the river can increase substantially. Communities adjacent to the river experience seasonal HEC, and in response, GNP managers have constructed beehive fences with the aim of reducing conflict levels. These fences consist of a series of suspended beehives, set 7 m apart and linked by rope. Elephants cannot cross the barrier without disturbing the bees (King, 2019; King et al., 2011, 2017). In GNP, the number of beehives per fence varies from 1 to 12, and the fences are not continuous. Fences are located along the southern bank of the Pùngué River in places that elephants have used to move into community areas in the buffer zone. The fences are distributed around eight communities subject to high levels of HEC (Figure 1), with more fences in areas of higher conflict. Park rangers and community members also use reactive and traditional measures, including fireworks and drums, to scare elephants away if they are detected close to agricultural fields.

### Movement data

We used remote injection to chemically immobilize 10 lead females from different family groups and 10 males (Appendices S1–S3). The females were found, darted, immobilized, and collared inside the park. Eight of the 10 males were found and collared in the buffer zone. We fitted model‐AWT IM‐SAT (10 D cells) GPS collars on females and model‐AWT Iridium‐SAT (12 D cells) on males. We collected hourly GPS location data from 2019 to 2022. All procedures for immobilizing, handling, and collaring elephants were approved by the University of Kent Animal Ethical Review Committee (7‐PGR‐20/21).

### Barrier data

All statistical analyses were conducted using R 4.2.2 (R Development Core Team 2021), and spatial data were created using QGIS 3.26 (QGIS Development Team, 2022). The beehive fence barrier GIS layer was created using point data recorded during installation. An additional 5 m was added to the end of each fence to account for the effect of the fence beyond the location of the final beehive. The river barrier GIS layer was based on a layer obtained from Mozambique's National Cartography database. This original layer had a very high spatial resolution, which substantially increased processing time without improving subsequent analyses. So, we used the QGIS simplify tool to ensure each point along the line was >200 m apart (Xu et al., 2021). We then divided the simplified layer into 300‐m segments for analyses.

### HEC data

We extracted HEC data for 2018–2021 from the GNP's Conservation Department rapid response database. These data were collected by rangers and consisted of GPS coordinates, recorded using Garmin 10 Etrex devices, and incident location, type, and severity (e.g., number of people killed or injured, crop damage, and infrastructure damage).

### Barrier behavior analyses

To identify and quantify barrier behaviors of elephants, we used the BaBA package in R (Xu et al., 2021, 2023). The BaBA (barrier behavioral analysis) is a temporal spatial approach used to examine individual behavioral responses to linear barriers (Xu et al., 2021). Barrier behavior is categorized as normal (no unusual movement or a quick cross of the barrier), altered (bounce, animal approaches the barrier and then moves away without crossing; back and forth, animal approaches the barrier and moves a short distance up and down it multiple times; trace, animal approaches the barrier and then moves along it in parallel), or trapped (animal is surrounded by a barrier system and cannot get out). We did not consider the trapped response because both beehive fences and the river are linear features, so elephants were always able to move away from them.

To parameterize the BaBA model, we ran a sensitivity analysis to determine the buffer distance on either side of the barrier that if an elephant's movement pathway was within it, it would be counted as an encounter event. To find the optimal buffer distance on which to base this designation of an encounter, where the optimal is defined as the distance at which the number of quick‐cross events either begins to decrease or has a <1% increase (Xu et al., 2021), we ran the BaBA model at 20‐m increments from 50 to 500 m for the beehive fences and at 50‐m increments from 50 to 800 m for the river. Finally, we ran the BaBA with the elephant GPS collar data and the barrier GIS layers to classify each of these encounter events into one of the six barrier behaviors. For each barrier encounter event detected by the model, we manually specified the direction the elephant was travelling (i.e., whether they moved into GNP from the park buffer or vice versa).

### Statistical analyses

We used a chi‐square test to determine whether the elephants showed differing responses when encountering either beehive fences or the river. We compared the incidence of average movement, quick cross, bounce, back and forth, and trace responses.

We estimated the permeability of the two barrier types with the baranking function from the BaBA package (Xu et al., 2021, 2023). This calculates a barrier permeability index by comparing the ratio of nonnormal movement to total encounters for each animal, weighted by their number of encounters with the barrier and scaled from 0 to 1 (Xu et al., 2021).

We investigated the efficacy of the barriers in altering elephant movement behavior. Our analysis included factors associated with crop‐raiding behavior and risk aversion, which is known to differ between males and females and to depend on seasonal crop availability (Branco et al., 2019; Chiyo et al., 2011; Wittemyer et al., 2013). We did this by developing a generalized linear mixed model (GLMM) with binomial distribution. Encounters categorized as normal behaviors were coded as 0. Altered behaviors were coded as 1. The global model included barrier type (beehive or river), sex, direction of altered movement (inside to outside the barrier or outside to inside), and season (dry or wet) as fixed variables. Interactions were barrier and sex, barrier and direction, barrier and season, season and sex, and sex and direction. We included elephant identity as a random variable and conducted the analyses with the glmer function in the lme4 package. We standardized and centered all fixed variables to have a mean of 0 and an SD of 0.5 (Cade, 2015), which put them at the same scale and allowed direct comparison of effect sizes. We verified that there were no collinearity problems among any pair of predictors. Variables were considered highly collinear if *r* ≥0.7 or GVIF ≥5 (Zuur et al., 2009). We then applied the dredge function to the global model with the MuMIn package (Barton, 2009), which produced a set of all possible model outcomes. We selected the best model based on the Akaike information criterion (Burnham & Anderson, 2004).

To evaluate spatial changes in reported HEC incidents before and after the installation of the beehives, we calculated a kernel density estimation with the Heatmap tool in QGIS. We used a 5‐km radius and 100‐m resolution to create density rasters for the periods before (2018 and 2019) and after (2020 and 2021) the implementation of the beehive fences.

## RESULTS

### Behaviors during encounters with beehive fences and the river

We recorded 186,165 GPS locations for male elephants and 166,105 GPS locations for females. Optimal buffer distances varied between the two barriers: 400 m for the river and 280 m for the beehive fences. The combined length of the beehive fences (7.7 km) was much shorter than the length of the river (76 km), but the mean elephant encounter rate was much higher for the beehives fences (442.5 per km) compared with the river (79.6 per km).

**FIGURE 2:**
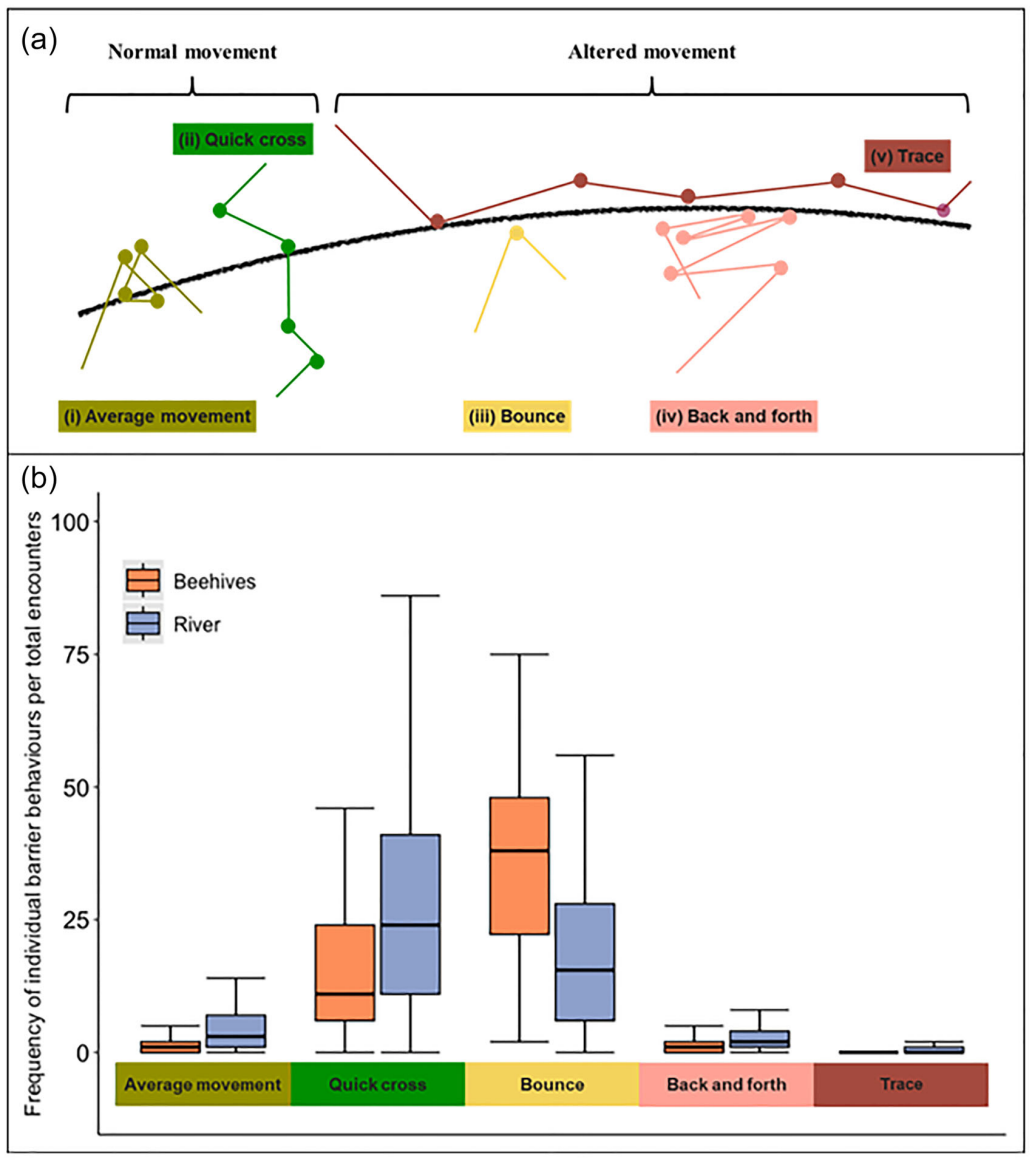
Frequency of behaviors of elephants (*n* = 20) in response to beehive fences and a river (average movement, animal does not change its pattern of movement in a significant manner; quick cross, animal moves quickly through the barrier; bounce, animal approaches the barrier and then moves away without crossing; back and forth, animal approaches the barrier and moves a short distance up and down it multiple times; trace, animal approaches the barrier and then moves along it in parallel; bars, interquartile range; horizontal line in bars, median; whiskers, minimum and maximum nonoutlier values). Panel (a) is adapted from Xu et al. (2021).

Behaviors registered upon encountering barriers were significantly associated with the barrier type (*χ* ^2^_4_ = 1003.17, *p* < 0.001) (Figure 2; Appendices S4 & S5). Quick cross and bounce were the most common behaviors, but quick cross was higher than expected when elephants encountered the river, and bounce was higher than expected when encountering beehive fences. Elephants tended to show high levels of normal behavior when encountering the river. When their behavior was altered, they tended to exhibit back‐and‐forth or trace behavior (Appendix S5). Conversely, when encountering the beehive fence, the bounce behavior was substantially more common than any other behavior (Appendix S5). The permeability of the barriers varied with type and location. Beehive fences were less permeable on average, and both barriers were less permeable along the western boundary of GNP (Appendix S5).

### Altered behaviors models

**FIGURE 3:**
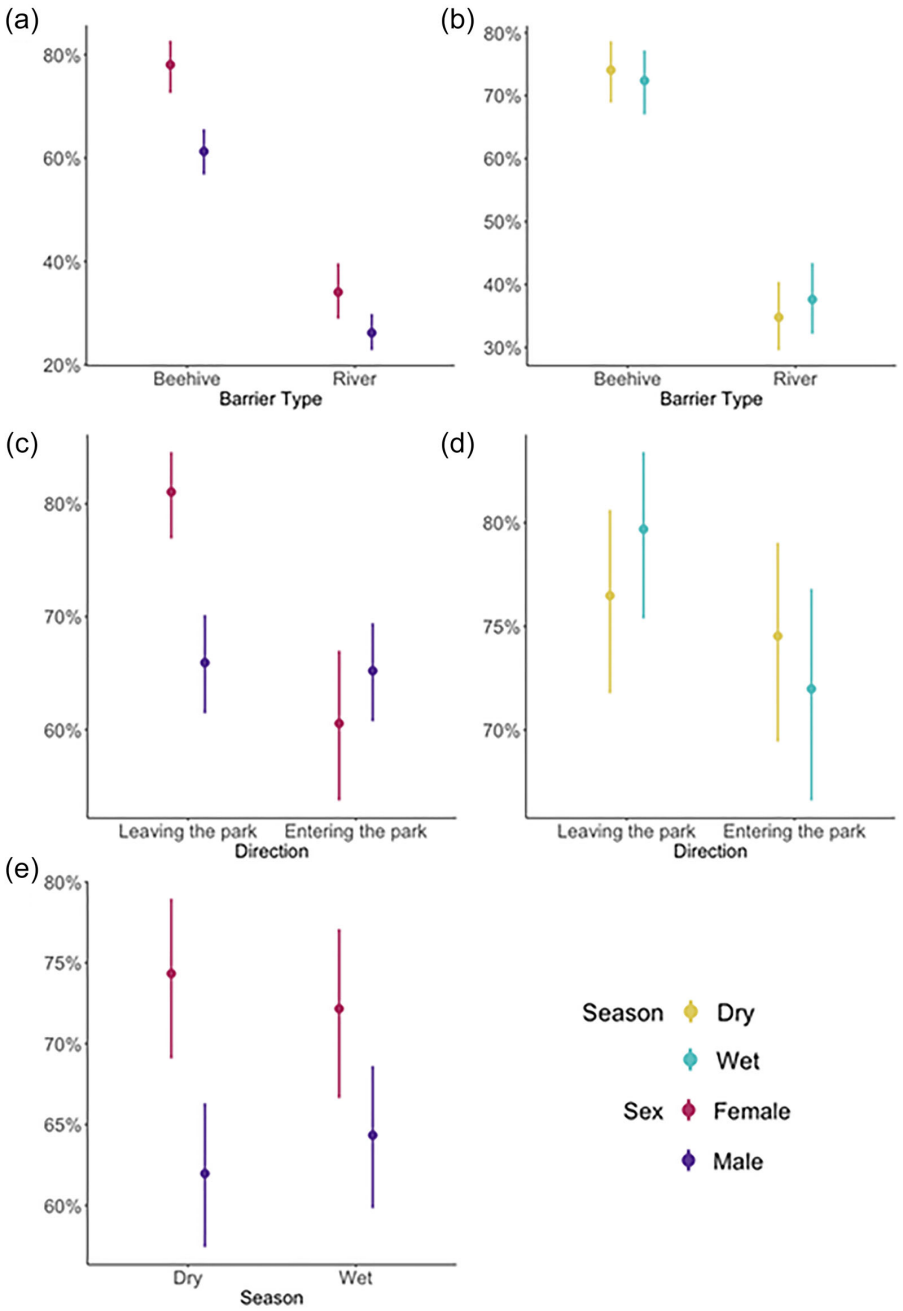
Modeled percentage of incidents where elephants changed their movement in response to beehive and river barriers by (a) sex, (b) season, (c) sex and direction of altered movement (from inside to outside and outside to inside the barrier), (d) season and direction of altered movement (from inside to outside and outside to inside the barrier), and (e) interaction between season and sex.

The top model for altered movement behavior included barrier type, direction of altered movement, sex of the elephant, and season (conditional *R* ^2^ = 0.074) (Table 1). There were significant interactions between barrier type and sex, direction of altered movement and sex, and season and sex (Table 1). Elephants had a higher probability of altered movement in relation to beehive fences than the river, and this difference was particularly strong for female elephants (Figure 3c). Females had a higher probability of altered movement when attempting to move from the park into community areas, compared with attempting to move from the community areas into the park (Figure 3d). However, there was no difference in the probability of altered movement for males in relation to their direction of travel (Figure 3d). The difference in probability of altered behavior between male and female elephants can also be seen across the seasons (Figure 3e). Males were much less likely to have altered behavior in the dry season, when crops are most abundant, compared with females (Figure 3e).

**TABLE 1:** Standardized generalized linear mixed model parameter estimates in a study of elephant responses to conflict mitigation fencing, for the model that best explains variation in altered elephant movement.

Variable	*β* coefficient^a^	2.5% to 97.5% CI	*p*
Intercept	2.043	−0.023 to 0.656	<0.001
Barrier river	−0.244	−0.49 to −0.077	<0.001
Direction	−0.987	−1.522 to −1.021	<0.001
Season	−0.176	−0.4 to 0.034	ns
Sex	−1.514	−1.882 to −1.052	<0.001
Barrier: season	0.218	−0.115 to 0.257	0.023
Barrier: sex	0.667	0.27 to 0.7	<0.001
Direction: season	−0.327	−0.357 to 0.026	<0.001
Direction: sex	1.128	1.216 to 1.725	<0.001
Season: sex	0.33	0.105 to 0.543	0.003

Abbreviation: ns, not significant.

^a^ Rate of change in the response per 1 SD change in the predictor.

### Patterns of HEC

**FIGURE 4:**
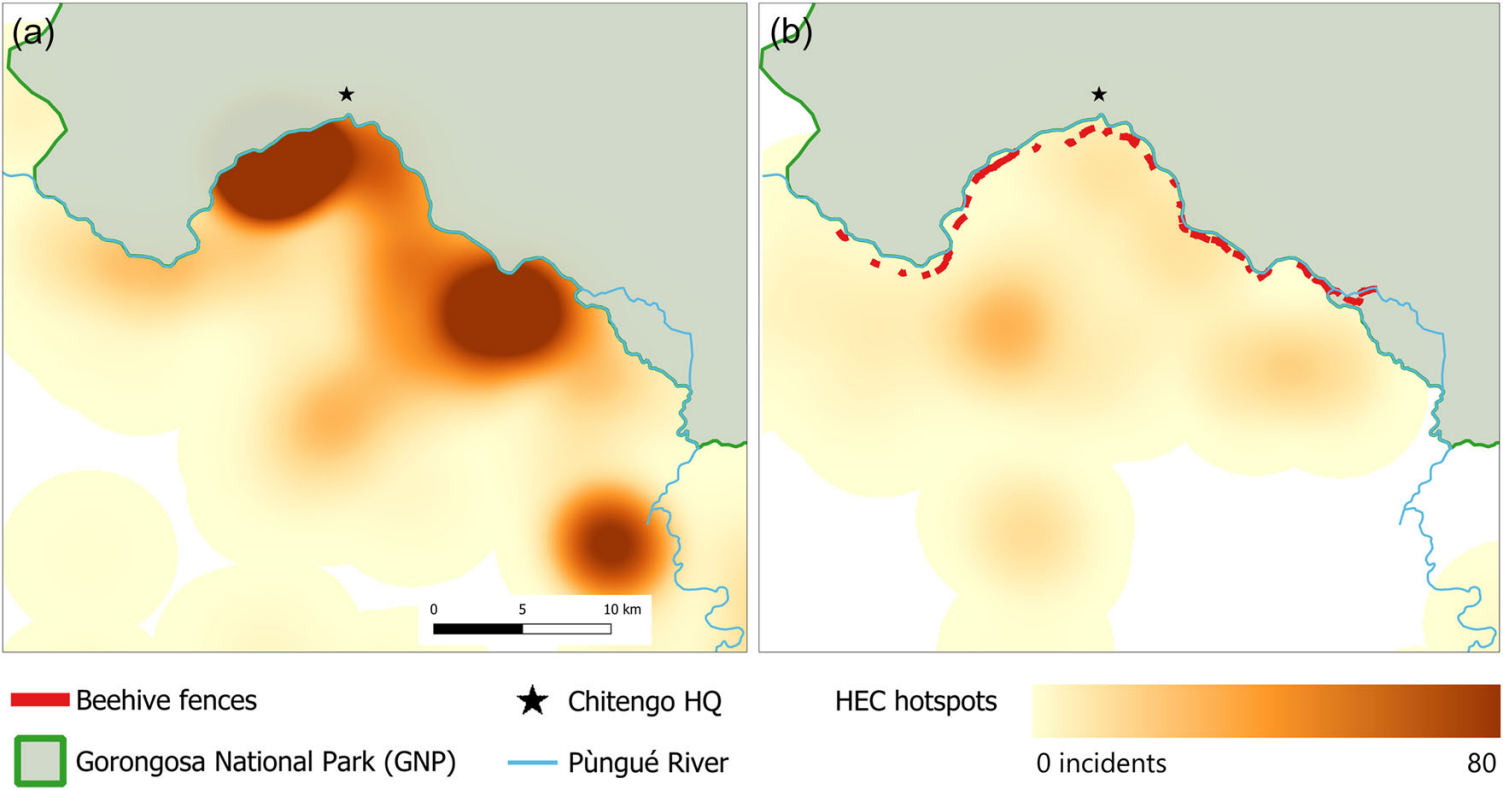
Conflict incident locations (a) before (2018–2019) and (b) after (2020–2021) the installation of beehive fences (HQ, park headquarters; HEC, human–elephant conflict).

Although the total number of HEC incidents declined after the beehive fences were installed, there was an increase in the number of severe incidents. In 2018 and 2019, there were no recorded deaths, 1 person was injured, and there were 1132 conflict incidents related to crop and infrastructure damage (i.e., a mean of 566 incidents per year). In 2020 and 2021, there were two recorded deaths, 6 people injured, and 235 conflict incidents related to crop and infrastructure damage (i.e., a mean of 117.5 incidents per year). The spatial distribution of HEC incidents also shifted (Appendix S6). More were reported farther south, away from the GNP boundary, and the mean distance of the closest crop and infrastructure damage incidents from the park boundary increased from 0.98 to 1.97 km (Figure 4).

## DISCUSSION

We used a new approach focused on individual behavioral responses to potential movement barriers (Xu et al., 2021) to evaluate the efficacy of an HEC mitigation intervention. We found elephant responses varied based on barrier type, sex, direction of travel, and season elucidating the mechanisms that underpin observed changes in HEC post intervention. Our results are consistent with previous work that suggests beehive fences effectively reduce overall records of HEC. However, by investigating landscape‐level changes in patterns of HEC, we found that although overall numbers of reported incidents decreased, there were shifts in the severity and spatial patterns of HEC incidents, which could have otherwise been missed and would have important implications for the success of HEC mitigation interventions.

### Elephant movement behavior types in response to linear barriers

We documented the different behaviors shown by elephants when encountering the linear barriers, with most of the recorded behaviors consisting of quick cross or bounce. Elephants reacted differently to the two barrier types; 35.9% of encounters with the river resulted in altered movement compared with 69.3% with the beehive fences. This difference was probably because the GNP elephant population has encountered the river regularly for many years and elephants cross all but the largest and deepest of rivers (Naidoo et al., 2022). In contrast, the beehive fences were erected relatively recently, with the express aim of altering elephant movement patterns. Other studies also show that natural barriers have less impact than fences on animal movement for a range of mammal species (Bischof et al., 2017; Cozzi et al., 2013; King et al., 2011; Wilkinson et al., 2021; Xu et al., 2023). Our results suggest the beehive fences are achieving their goal, with many examples recorded of elephants approaching the barrier and then moving away from it, back toward the park.

We also found that barrier permeability varied along the length of the river and between the different beehive fences. For the river, this was likely due to geophysical characteristics; elephants find it easier to cross shallow river sections and areas with relatively low riverbanks (Branco et al., 2020). The variation in permeability of the beehive fences may in part be due to the behavior of the bees. Colonies can sometimes leave their original hives, and it takes time for the people managing the fences to replace them, leaving the hives temporarily empty (personal observation). Unoccupied beehive fences repel elephants, but the effect is weaker than observed for occupied fences (Branco et al., 2020).

### Factors associated with altered elephant movement behavior

Individual elephants had different frequencies of encounters with the two barrier types and also different behavioral responses (Appendix S5); females had fewer interactions than males. This difference may be partly explained by the selection of individuals for collaring. The collaring of males focused specifically on individuals that were known to damage crops, meaning they necessarily came into contact with the barriers. Females were selected with a focus on ecological monitoring and so were more likely to range within the park. However, all the elephants interacted with the barriers multiple times during the study period and, although there were some sex differences, the broader trends were quite consistent. Individual effects were accounted for in our analyses, and our results are in accordance with a study that showed that HEC barriers can have different effects across individuals (Mumby & Plotnik, 2018).

We found a significant interaction between barrier type and sex. Females had a higher probability of altered behavior for both barrier types. Chelliah et al (2010) also found that HEC mitigation fences had a greater deterrent effect on females than on males at a site in India. This may be due to higher levels of risk aversion (Chiyo et al., 2011; Wittemyer et al., 2013), which likely arises in part because females are part of family groups that include calves, and these younger, smaller individuals find it difficult to cross deep rivers (Poole & Granli, 2017) and may be particularly vulnerable to being stung by bees (King et al., 2017). Encountering a beehive fence may also have other indirect effects on family groups, such as high stress and potential separation of calves and mothers (Jakes et al., 2018). Males tend to travel alone or in small bachelor groups, so they are better able to cope with risky situations (Ahlering et al., 2011; Chiyo et al., 2011). Moreover, studies on other species where parental care is primarily done by one sex show that individuals of that sex avoid locations with a high potential for encountering people (Morrow et al., 2019; Oliveira et al., 2018).

We also found a significant interaction between the elephants’ direction of travel and their behavior. During the wet season, elephants were more likely to exhibit altered behavior when moving from inside the park into community areas when compared with the dry season. This may relate to the level of motivation the elephants have to move into the buffer zone because during the wet season there is abundant high‐quality food available in the park, whereas the crops growing outside the park are still maturing (Branco et al., 2019). The relative benefits of being inside the park may also explain the sex differences we found in relation to the direction of travel. Females were much more likely to exhibit altered movement behavior when attempting to leave the park and were less likely to exhibit it when returning (Figure 3). This may be because the greater safety of being within the borders of GNP outweighs the potential risks associated with crossing the barriers.

### Impacts of barriers on HEC

In the years after the beehive fences were erected, there was a substantial decrease in the reported number of HEC incidents. However, although the absolute numbers of incidents decreased, there were more incidents that resulted in human injury or death. This is supported by community reports that elephants used to raid crops at night and then immediately return to GNP but now stay in the buffer zone for longer periods, hiding in forest patches and sometimes raiding crops during the day. This results in elephants being closer to people for more time, increasing the potential for dangerous encounters. This also explains our finding that the locations of conflict incidents shifted farther from the park boundary. This change in HEC distribution is consistent with findings from other study areas where fencing displaced HEC to new areas (Osipova et al., 2018). This may have significant impacts on communities that have not previously had experience with HEC and therefore have lower tolerance levels and lower engagement with (and therefore benefits from) mitigation measures, such as beehive fences.

Human–wildlife conflict mitigation is an important conservation issue, and developing effective mitigation measures is key. Our results show the need to properly monitor how these mitigation interventions affect animal behavior. Previous studies have focused on direct impacts, such as whether levels of crop raiding have dropped (Branco et al., 2020; Davies et al., 2011; King et al., 2017; Scheijen et al., 2019). However, it is important to also take a broader, landscape view to evaluate whether conflict has decreased more widely or moved to new areas. This monitoring needs to be long term to capture animal behavioral responses and adapt mitigation strategies if individuals become habituated (Fernando et al., 2008). These efforts must be tailored to local conditions (Thompson & Homewood, 2002) and codeveloped with communities to identify workable mitigation strategies that will decrease conflict and foster coexistence (Nog et al., 2015).

## Supporting information




Supporting Information
