# Supplementary material for: Elephant barrier behaviors in response to conflict mitigation fences
Source: Conserv Biol. 2026 Mar 19;40(2):e70258. doi: 10.1111/cobi.70258 (PMC13036287; doi:10.1111/cobi.70258)
Supplement: Supplementary file 1 — Supporting Information [file COBI-40-e70258-s001.docx]

# Supplementary material for ‘Elephant barrier behaviors in response to conflict mitigation fences’ by Gonçalves et al.

# Appendix 1: Identifying individuals for GPS collaring

Our analysis was based on ongoing work to monitor and understand the behavior and impacts of savanna elephants (Loxodonta africana) in and around Gorongosa National Park. This varies markedly between females and males (Moss and Poole 1983; Archie et al. 2006). Female elephants live in family groups, composed of an adult female or females, together with their calves. These groups show high levels of association and affiliative behavior, so lead females are highly unlikely to be without their family unit. This makes tracking female elephants with GPS collars relatively straightforward, as it only requires collaring one lead female from each family groups. To identify suitable females to dart while surveying from a helicopter, we applied the following criteria in consultation with veterinary and wildlife capture specialists:

- Age and pregnancy. We looked for females between 25-60 years old. We estimated their age from the air based on physical characteristics, such as shoulder height and back length. We avoided any female that was observed to be pregnant to reduce the risk for the animal.
- Behavior. Each family groups contained an individual, or individuals, that stood out as lead females based on their behavior in the presence of the helicopter. In particular, we carefully observed each group to identify females that led the rest of the group away from the helicopter, led the group to shelter and/or moved away from the group to try and charge the helicopter.
- Presence and age of calf. We did not collar lead females with a calf younger than 4 years old. This is because such calves generally stay by their mother’s side, which would cause additional stress during the darting process. It could also lead to the rest of the family group trying to rescue the calf, making it unsafe for both the collaring team and the elephants.

Male elephants in Gorongosa tend to be solitary or found in small groups, while also being associated with family groups when seeking mating opportunities. In particular, groups of male elephants have established in the areas of Chimuto, Nhartuzo and Mussapassua during and after the floods caused by Cyclone Idai in March 2019. These groups are often involved in human-elephant conflict incidents around the national park and so were a particular focus of GPS collar monitoring. Older elephants within a male group can be considered the leader, as they are normally followed by the younger males, although these associations can be short-term. Thus, to identify suitable males to dart while surveying from a helicopter, we focused on solitary males and both the oldest/largest and youngest individuals within a group.

## Appendix 2: Elephant collaring procedure

To deploy the GPS collars (Model AWT IM-SAT, Africa Wildlife tracking, Pretoria, South Africa), we worked primarily at the coolest hours of the day, i.e. early morning or late afternoons. Using a helicopter, we searched the area for appropriate targets for darting (as described above). Once a viable target was identified, we drove it and its associated group towards a clearing in the vegetation on firm ground. This was of outmost importance, as it avoided the need for the darted elephant to expend additional effort walking through water or thick vegetation, which can accelerate the effect of the tranquillizer.

Once in the selected area, the target elephant was chemically immobilized from the helicopter using a remote injection into one of the animal’s muscle blocks. The drugs injected were a combination of Thianhil and Azaparone, with the dosage depending on the approximate age and size of the animal (Table S1). It would take an average of eight to 12 minutes for the target to fell asleep. As soon as the elephant fell asleep, the team quickly intervened. It was crucial that the elephant was positioned in the best possible location for the collaring operation, accounting for both the safety of the elephant and the collaring team. On occasions when the animal fell asleep in an upright sitting position, the team would try to push it onto one of its sides to avoid pressure on its sternum and facilitate easy breathing. In cases where the team was unable to push the elephant, they instead focused on finishing the collaring process as quickly as possible. Simultaneously, the team worked to keep the targeted animal isolated by gently driving the rest of the group back towards nearby thicker vegetation.

While the GPS satellite collar was being attached and set, we monitored the darted elephant’s reaction to disturbance from the helicopter and its vital signs by regularly checking its breathing and eyes. We also collected blood and fecal samples and estimated its age and body condition. The body condition score is a subjective assessment of subcutaneous body fat, based on visual evaluation of muscle tone and key skeletal elements (Morfeld *et al.*, 2014). The method is based on a numeric scoring system defined in Morfeld *et al.* (2014, 2016), which gives scores ranging from 1 for very thin to 5 for very fat.

To prevent any infections, the team administrated penicillin in any observed wounds in the darted animal’s body, including in the spot where the dart entered the muscle. Once the collar was successfully attached, Trexonil was given to revert the tranquilizer and wake the animal. The team then returned to the helicopter and stayed airborne but close to the animal for an average of ten minutes, checking the animal’s reaction to the administered drug and that the collar was correctly fitted. On most occasions we observed that the rest of the group waited nearby, vigilant and vocalizing, until the collared elephant made its way to reunite with them. We closely monitored the behavior of the individual and its group after the collaring exercise, using the location data produced by the GPS collar. We found that the darted elephant would not move far for a few hours until the effects of the drugs wore off, but then returned to showing expected movement patterns.

Table S1: The amount of each immobilization drug used during the 2018 and 2019 collaring exercise that took place in Gorongosa National Park and its buffer zone. The amounts were based on the sex of each elephant (n=20).

| Sex | Thianil (mg) | Azaperone (mg) | Trexonil (mg) | Penicillin (ml) |
| --- | --- | --- | --- | --- |
| Females | 10 | 40 | 10 | 10 |
| Males | 10 | 40 | 15 | 10 |

## Appendix 3: Interpreting the collared elephant data

The amount of data collected on each elephant varied, based in part on when each elephant was first collared and whether it was recollared (Table S2). For example, one male and one female died after a year of data collection and five males dropped their collars. For the remaining 13 elephants, their collars were removed and replaced with new collars during the study period. The behavior of the collared lead females was likely to be representative of their family groups, for reasons described above. However, for males found in a particular male group when darted, it is unlikely that these associations continued for the long-term. This is especially for males involved in human-elephant conflict, as monitoring staff noted that young bulls would form small groups in the day but join larger groups at night when crop-raiding.

Table S2: Collared elephants’ details observed during the collaring exercise and its fate during data collection period from 2018 to 2021 in Gorongosa National Park.

| ID | Age | BCS | Group Size | Sex | Date start | Fate |
| --- | --- | --- | --- | --- | --- | --- |
| F1-Patricia | 25 | 3 | 7 | F | 05.11.2018 | Recollared |
| F2-Maria | 30 | 3 | 7 | F | 09.11.2018 | Mortality |
| F3-Milana | 36 | 4 | 12 | F | 09.11.2018 | Recollared |
| F4-Grainee | 35 | 3 | 7 | F | 05.11.2018 | Recollared |
| F5-Betty | 55 | 4 | 9 | F | 09.08.2018 | Recollared |
| F6-Anne | 35 | 4 | 12 | F | 09.08.2018 | Recollared |
| F7-Julianne | 49 | 3 | 5 | F | 09.08.2018 | Recollared |
| F8-Valente | 39 | 3 | 40 | F | 07.08.2018 | Recollared |
| F9-Dora | 45 | 3 | 12 | F | 08.08.2018 | Recollared |
| F10-Megan | 40 | 3 | 10 | F | 08.08.2018 | Recollared |
| M1-Brett | 40 | 4 | 5 | M | 02.08.2019 | Dropped collar |
| M2-Bryan | 32 | 3 | 5 | M | 02.08.2019 | Recollared |
| M3-Louis | 45 | 4 | 10 | M | 02.08.2019 | Recollared |
| M4-Matt | 30 | 3 | 1 | M | 03.08.2019 | Dropped collar |
| M5-Mutemba | 40 | 4 | 17 | M | 06.08.2019 | Dropped collar |
| M6-Pingo | 45 | 4 | 3 | M | 06.08.2019 | Recollared |
| M7-Quentin | 45 | 4 | 17 | M | 06.08.2019 | Dropped collar |
| M8-SP | 60 | 4 | 1 | M | 08.08.2019 | Mortality |
| M9-Tonecas | 35 | 4 | 10 | M | 10.08.2019 | Recollared |
| M10-Tyler | 30 | 3 | 1 | M | 08.08.2019 | Dropped collar |

## Appendix 4: Barrier Behavior types analysis results


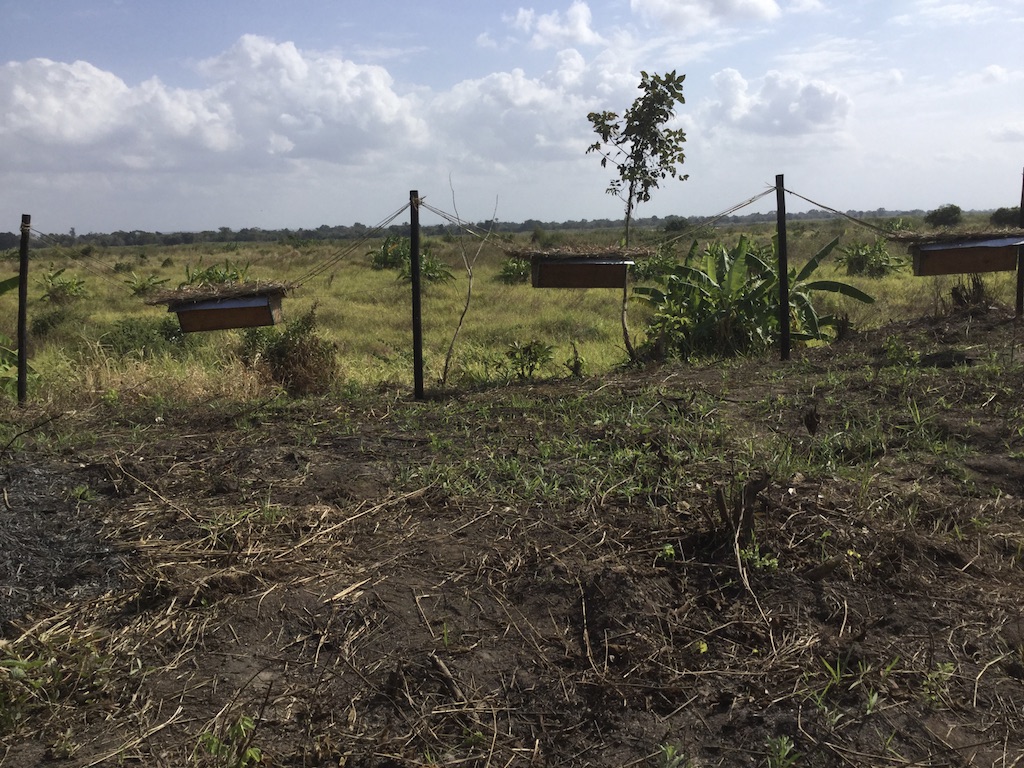


Figure S1: A photograph of a beehive fence set up in Gorongosa National Park buffer zone.

Table S3. Barrier behavior for beehive fences and river showing how the observed frequencies differ from the expected behaviors.

| Barrier type | Behavior grouping | Behavior type | Number of incidents | Difference from expected (%) | χ2 |
| --- | --- | --- | --- | --- | --- |
| Beehive | Normal | Average Movement | 76 | -54 | 48.04 |
|  |  | Quick cross | 900 | -39 | 218.64 |
|  | Altered | Bounce | 2128 | 48 | 336.86 |
|  |  | Back and forth | 72 | -29 | 8.49 |
|  |  | Trace | 7 | -59 | 6.11 |
| River | Normal | Average Movement | 354 | 34 | 29.92 |
|  |  | Quick cross | 2920 | 24 | 136.19 |
|  | Altered | Bounce | 1606 | -30 | 209.83 |
|  |  | Back and forth | 192 | 18 | 5.29 |
|  |  | Trace | 38 | 37 | 3.81 |

## Appendix 5: Barrier Behavior analysis of individual elephants

Table S4: Individual variability of elephant encounters and movement behaviors for beehive fences and river barriers (Normal movements = average movement or quick cross; Altered movements = Bounce, Back and forth or Trace as defined by Xu et al., 2021).

|  |  |  | Beehives |  |  | River |  |
| --- | --- | --- | --- | --- | --- | --- | --- |
| Sex | ID | Total | % Normal movements | % Altered movements | Total | % Normal movements | % Altered movements |
| Females | F1 | 30 | 36.67 | 63.33 | 138 | 60.87 | 39.13 |
|  | F2 | 1 | 0 | 100 | 2 | 50 | 50 |
|  | F3 | 17 | 5.88 | 94.12 | 42 | 50 | 50 |
|  | F4 | 1 | 0 | 100 | 12 | 33.33 | 66.67 |
|  | F5 | 263 | 13.69 | 86.31 | 547 | 50.46 | 49.54 |
|  | F6 | 9 | 22.22 | 77.78 | 103 | 48.54 | 51.46 |
|  | F7 | 1 | 100 | 0 | 20 | 30 | 70 |
|  | F8 | 3 | 0 | 100 | 9 | 22.22 | 77.78 |
|  | F9 | 106 | 10.38 | 89.62 | 198 | 59.6 | 40.4 |
|  | F10 | 147 | 14.29 | 85.71 | 287 | 52.96 | 47.04 |
| Males | M1 | 401 | 34.66 | 65.34 | 559 | 69.41 | 30.59 |
|  | M2 | 398 | 30.65 | 69.35 | 484 | 63.43 | 36.57 |
|  | M3 | 337 | 45.7 | 54.3 | 414 | 72.46 | 27.54 |
|  | M4 | 43 | 18.6 | 81.4 | 128 | 62.5 | 37.5 |
|  | M5 | 34 | 29.41 | 70.59 | 136 | 63.97 | 36.03 |
|  | M6 | 354 | 28.25 | 71.75 | 378 | 67.46 | 32.54 |
|  | M7 | 84 | 27.38 | 72.62 | 252 | 52.38 | 47.62 |
|  | M8 | 4 | 25 | 75 | 31 | 48.39 | 51.61 |
|  | M9 | 423 | 32.39 | 67.61 | 592 | 73.65 | 26.35 |
|  | M10 | 527 | 37.76 | 62.24 | 778 | 71.98 | 28.02 |

## Appendix 6: Elephant movement before and after construction of fences


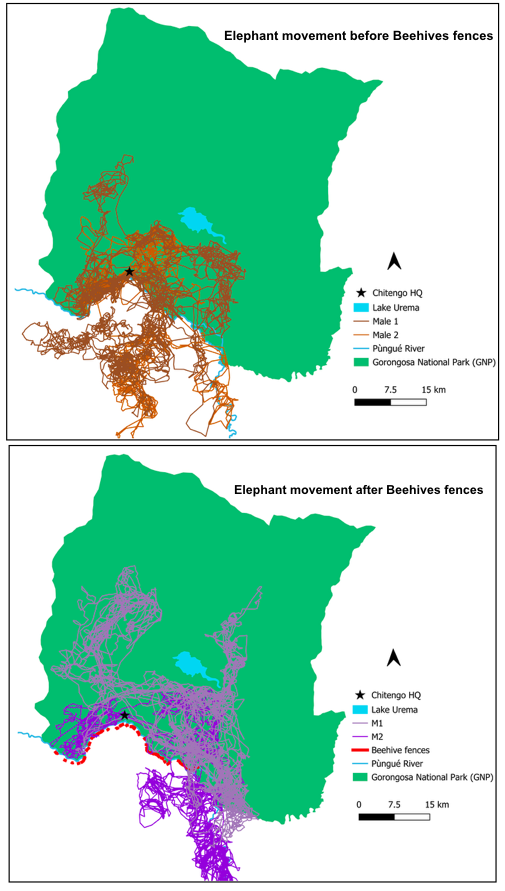


Figure S2: An illustration of GPS-tracks (one river crossing section before/after beehive fences in Gorongosa National Park.

## References

Archie, E.A., Moss, C.J., Alberts, S.C., 2006. The ties that bind: Genetic relatedness predicts the fission and fusion of social groups in wild African elephants. Proceedings of the Royal Society B: Biological Sciences **273**, 513–522.

Moss, C.J. and Poole J.H. (1983). Relationships and social structure in African elephants. In Primate social relation- ships: an integrated approach: 315–325. Hinde, R.A. (Ed.). Oxford: Blackwell Scientific Publications.

Morfeld, K.A. et al., (2014) ‘Development of a Body Condition Scoring Index for Female African Elephants Validated by Ultrasound Measurements of Subcutaneous Fat’. PLoS ONE 9(4): e93802. doi: 10.1371/journal.pone.0093802

Morfeld K.A. et al., (2016) ‘Assessment of Body Condition in African (Loxodonta africana) and Asian (Elephas maximus) Elephants in North American Zoos and Management Practices Associated with High Body Condition Scores’. PLoS ONE 11(7): e0155146. doi:10.1371/ journal.pone.0155146

Xu, W. *et al.* (2021) ‘Barrier Behaviour Analysis (BaBA) reveals extensive effects of fencing on wide-ranging ungulates’, *Journal of Applied Ecology*, 58(4), pp. 690–698. Available at: https://doi.org/10.1111/1365-2664.13806.
